# How to evaluate lifelong learning skills of healthcare professionals: a systematic review on content and quality of instruments for measuring lifelong learning

**DOI:** 10.1186/s12909-024-06335-9

**Published:** 2024-12-05

**Authors:** Monica H. M. Verkooijen, Anne A. C. van Tuijl, Hiske Calsbeek, Cornelia R. M. G. Fluit, Petra J. van Gurp

**Affiliations:** 1 Department of Psychiatry, Radboud University Medical Center, Nijmegen, The Netherlands; 2 Learning and Innovation Centre, Avans University of Applied Sciences, Breda, The Netherlands; 3 IQ Health Scientific Department, Radboud University Medical Center, Nijmegen, The Netherlands; 4 Department for Research on Learning and Education, Radboudumc Health Academy, Nijmegen, The Netherlands; 5 Department of Internal Medicine, Radboud University Medical Center, Nijmegen, The Netherlands

**Keywords:** Lifelong learning, Healthcare professionals, Instruments, Systematic review, Measurement properties, Content

## Abstract

**Background:**

Having lifelong learning skills is a necessity for healthcare professionals. To evaluate these skills, sound instruments are needed. Those working in healthcare of medical educating looking for a suitable instrument to evaluate lifelong learning (LLL) skills in healthcare professionals are faced with a multitude of definitions and operationalizations of the concept of LLL. A systematic review was performed to analyze the methodological quality and content of instruments measuring LLL for professionals.

**Methods:**

A systematic search of literature published until October 2023 in the electronic databases MEDLINE, CINAHL, PsycINFO, ERIC, Sociological Abstracts, EMBASE, and Web of Science was conducted. English articles describing the development, validation or use of an instrument measuring lifelong learning for professionals working in a professional context were included. A data extraction form was developed to evaluate the content and methodological quality of the instruments. The STORIES statement was used to support reporting this review.

**Results:**

The search revealed 85 articles on 18 questionnaires, no qualitative instruments were found. The instruments covered a range of settings, for example the Jefferson Scale of Physician LLL (JSPLL) covered healthcare and the Effective LLL Inventory (ELLI) covered education. Most instruments provided evidence on content validity and internal consistency, other aspects of validity were examined less frequently. Furthermore, the way that LLL was defined varied greatly, also great variety in the constructs that were defined in the instruments was found. Most instruments lacked a clear description of how the dimensions and items were formalized.

**Conclusions:**

There is a need for LLL instruments that provide more evidence on their validity and put greater emphasis on the development of the definition and operationalization of LLL. Furthermore, there is a need for a better understanding of how to interpret and use the results of the instruments. It is recommended to take a critical look at these constructs before selecting an instrument. This will help all those involved in the training and working environment of healthcare professionals in evaluating lifelong learning skills in their context.

**Registration:**

PROSPERO registration number: CRD42019134804

**Supplementary Information:**

The online version contains supplementary material available at 10.1186/s12909-024-06335-9.

## Background

To meet the challenges of a rapidly evolving healthcare system, a workforce of flexible, resilient, and adaptive professionals is essential [1–3]. In this context, the ability to learn and adapt has become an essential skill [4, 5]. Lifelong learning (LLL), defined as the continuous, self-motivated pursuit of knowledge, values, and skills for personal and professional growth, is vital for healthcare professionals [5, 6]. This concept has gained widespread attention from institutions, associations, and regulatory bodies in healthcare globally [5–8]. Its importance is reflected in medical education, where programs are designed to foster LLL skills from undergraduate training to continuing medical education [2, 9].

LLL occurs in both formal and informal educational settings, including the workplace and social contexts [1, 2]. As a multifaceted concept, it involves skills like information-seeking, goal motivation, and self-directed learning [1, 7]. LLL has multiple definitions, stemming from a variety of approaches to frame LLL, each with its own conceptual focus [6] and ranging from easy-to-read definitions to complex definitions that include multiple other behavioral constructs [9]. Much like the multitude of LLL definitions, the literature describes a large variety of skills belonging to the lifelong learner, more or less based on theory, and more or less fitting a clear definition of LLL [7, 8].

This diversity of definitions and operationalizations complicates the evaluation of LLL. Evaluating LLL skills is crucial for healthcare professionals to reflect and improve. It also aids educators, curriculum coordinators, and policy makers in effectively supporting LLL development. On a broader scale, it helps create better learning environments and adjust learning activities to support professionals’ LLL skills.

Given the importance of LLL in healthcare, an overview of tools for evaluating LLL for professionals is needed. This review provides this overview, assessing the tools methodological quality, and examining how LLL is conceptualized and which skills are evaluated. Our rationale for not limiting the review to instruments specific developed for the healthcare stems from the assumption that LLL is a concept employed and measured in various contexts, and the medical profession can benefit from insights gained from its use in these diverse settings. The review addresses the following research questions:

1. What instruments are available for evaluating Lifelong Learning (LLL) skills among healthcare professionals?
2. What is the methodological quality of instruments assessing LLL skills in healthcare professionals?
3. How is LLL conceptualized and operationalized in instruments for evaluating LLL skills in healthcare professionals?

## Methods

### Study design

We performed a systematic review including articles published until October 2023. The PRISMA Checklist was used to report this study [10], see additional file 1. The COSMIN standards were used to develop the scoring form to assess the methodological quality of the included studies [11]. The protocol was registered on the PROSPERO register (CRD42019134804). The STORIES statement was used to support the reporting of this review.

### Data sources and search strategy

We conducted a systematic search in the following databases: MEDLINE, CINAHL, PsycINFO, ERIC, Sociological Abstracts, Embase, Web of Science. Keywords used were: 1) ‘Lifelong Learning’ and ‘continuing professional development or learning’ and 2) tools like conversation, interview, portfolio, survey, and questionnaire. Only English articles were included. Additional file 2 provides the full search strategy [see additional file 2]. A librarian with expertise in systematic reviews helped design the search strategy. The search was first conducted on April 10, 2019, and updated on May 28, 2020, and October 4, 2023. Reference lists of included articles were manually screened for additional studies.

### Eligibility criteria

We included studies that 1)were peer-reviewed, 2)focused on participants that are professionals working in a professional context, including trainees, residents, and postgraduates, 3) developed or used instruments measuring multi-faceted LLL. We excluded studies that 1)focused on a single aspect of LLL, 2)focused on participants not working in a professional context (e.g., undergraduates), 3)focused on (mandatory) continuing professional education, 4)evaluated educational programs or needs assessments.

### Screening process

All articles were imported into Endnote X9, and duplicates were removed in rounds. Two reviewers (AT or MV) initially screened over 12,000 articles based on title and abstract, resolving disagreements through discussion. The next 4000 articles were assessed by one primary researcher (AT or MV), with a random 10% checked by a second reviewer (CF). In the 2023 search update, this process was repeated. With 750 abstracts screened by both primary researchers, and 6000 more reviewed by one researcher (MV). Again a random sample of 10% was checked by a second reviewer (CF). All articles included based on title and abstract were then, if available, full-text screened by two researchers, at least one of these reviewers was MV (the full-text screening form is available on request). Any disagreement between reviewers or doubt about definite inclusion or exclusion was resolved by discussion.

### Data extraction

Data extraction was performed by two reviewers per article, with one primary reviewer (AT or MV) and another team member. A piloted data extraction form (available upon request) was used, consisting of five sections: (1) general information (e.g., year, country), (2) subjects and respondents, (3) methodological quality criteria, (4) instrument feasibility, and (5) instrument content. The second and third section were based on the COSMIN checklist [11]. We assessed the included studies on content validity, internal consistency, reliability, measurement error, structural validity, construct validity, responsiveness, and cross-cultural validity. Table 1 provides the definition of each measurement property. Each property was rated on a four-point scale (1 = inadequate to 4 = very good) [10], producing an overall methodological quality score. Feasibility focused on the number of questions and duration, while content analysis examined the instrument’s LLL conceptualization, origin, item types, practical uses, and related concepts. Disagreements were resolved through discussion.

**Table 1:** Description of each measurement property based on the COSMIN guidelines

Measurement property	Description
Content validity	Describes the extent to which the items of an instrument is representative of the construct to be measured.
Structural validity	Describes the extent to which scores of an instrument are representative of the dimensions of the construct to be measured.
Internal consistency	Describes the degree of the coherence of items of an instrument.
Cross-cultural validity	Describes the extent to which the items that were originally generated are applicable in a cultural different situation, such as ethnicity, gender or age groups.
Reliability	Describes the extent to which scores are overall consistent in repeated measurement and different conditions.
Measurement error	Describes the difference between a measurement and its true value, a systematic and random error.
Construct validity	Describes the extent to which the scores of an instrument are consistent with theoretical hypothesis and therefore represent if it’s indicative of the theoretical construct.
Responsiveness	Describes if an instrument is able to detect change over time in the measured construct

## Results

**Fig. 1:**
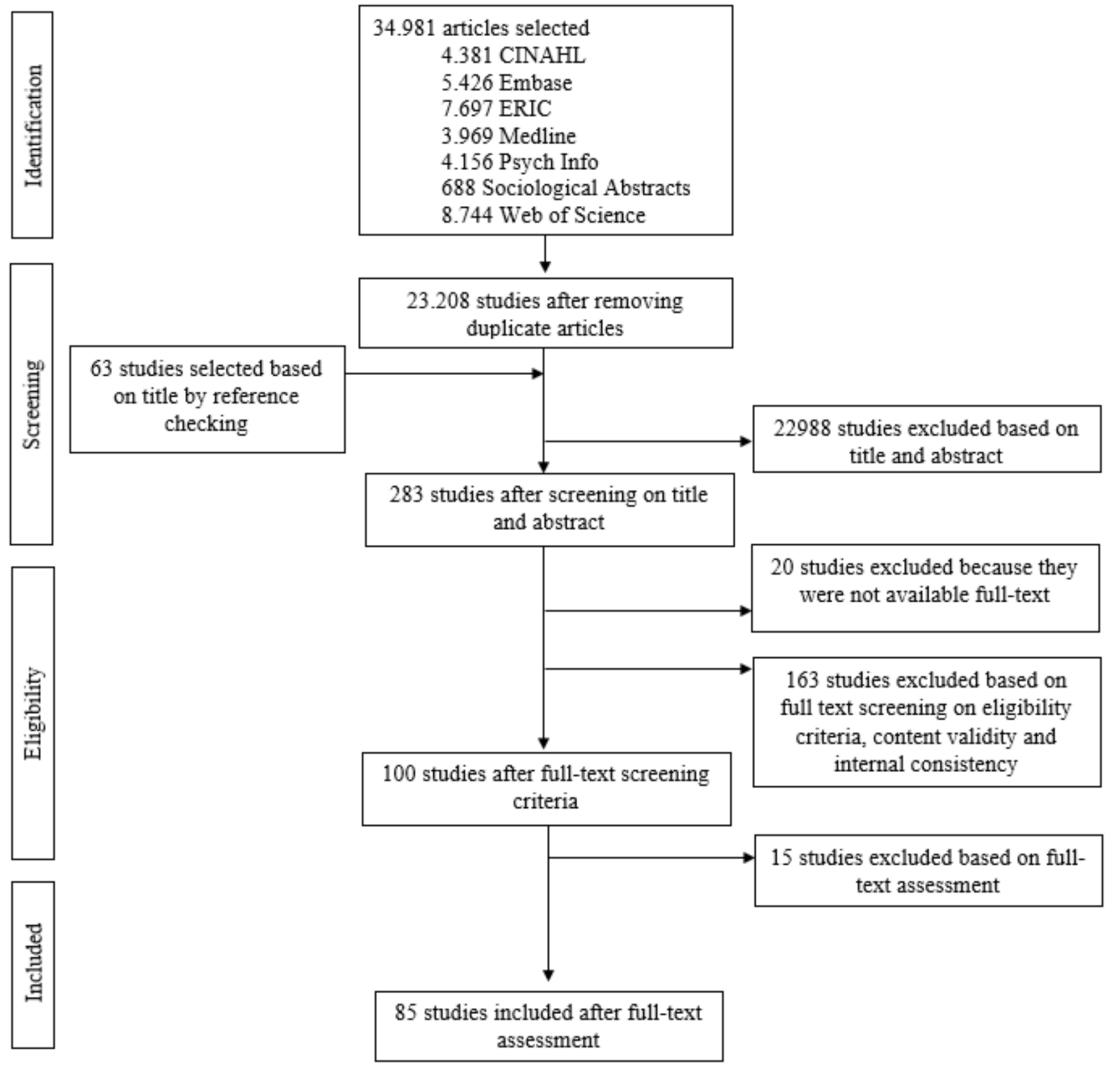
Flow of studies in a systematic review of instruments measuring lifelong learning for professionals

The searches yielded a total of 34,981 articles. After removal of duplicates, 23,208 studies were screened on title and abstracts, and 283 articles were selected for full-text reading. Out of these 283 articles, 100 were full text assessed, another 14 articles found through reference checking were full text assessed. Finally, 85 articles [7, 12–95] were included in this review. Eighteen articles focused on the development of a quantitative instrument measuring LLL, the remaining 67 focused on validation and/or used one of these instruments. Figure 1 presents the flowchart.

The characteristics of the 18 studies focusing on the development of an instrument measuring LLL for professionals are presented in Table 2.

**Table 2:** Key features and main characteristics of the 18 instruments measuring lifelong learning for professionals

	Authors	Name of the instrument	Abbreviation	Work setting & target population	Country	Instruments measure aim	Constructs of LLL instrument (*n* items)	Validated or used in:
1.	Oddi (1986)^a^ [12]	Oddi Continuing Learning Inventory	OCLI	*Education* Graduate students in Law, Nursing and Adult Education [12, 14] Registered nurses [13]	US [12–14]	Identifying self-directed continuing learners	1. Proactive/reactive learning drive [11] 2. Commitment/aversion to learning [7] 3. Cognitive openness/defensiveness [6] *N* = 24	13–14
2.	Livneh (1988) [15]	Characteristics of Lifelong Learners in the Professions	CLLP	*Human Service profession* Human Service professionals [15–17] Educators [18]	US [15–18]	Lifelong learner characteristic	1. Professional growth through learning [6] 2. Self-motivated achievement [5] 3. Educability [4] 4. Readiness for change [4] 5. Causation for learning participation [6] 6. Familial educational background [3] 7. Future orientations [4] *N* = 32	16–18
3.	Conti & Fellenz (1999) [19]	Self-Knowledge Inventory of LLL Strategies	SKILLS	*Diverse* Including educators, students, clerical workers, farmers, blue-collar workers, and homemakers [19] Nursing students [20]	US [19, 20]	Adult learning strategies	1. Metacognition 2. Meta motivation 3. Memory 4. Resource management 5. Critical thinking *N* = 18 (number of items per construct not specified)	20
4.	Hanson & DeMuth (1992) [21]	Characteristics of Lifelong Learners in the Professions scale	CLLPS	*Healthcare* Pharmacists [21, 22]	US [21, 22]	LLL characteristics	1. Intellectual curiosity [10] 2. Learning style [8] 3. Learning skills [7] 4. Family role model [3] 5. Social orientation to learning [3] 6. Professional enrichment [3] 7. Professional learning activities [6] 8. Leisure learning activities [8] 9. Perceptions of LLL [4] *N* = 52	22
5.	Hojat et al. (2003) [7]	Jefferson Scale of Physician LLL	JSPLL	*Healthcare* Physicians [7, 23, 26, 29, 31, 33, 35, 37–39, 41–44] Nurses [25, 26, 41, 42, 44] Psychologists [27, 44] Residents [28, 30, 34, 36] Medical Science staff [32, 44] Physical Therapists [40, 44]	US [7, 27–30, 36, 37, 39, 40] The Netherlands [23, 24] Uganda [25] South America [26] Canada [31, 34, 35] Iran [32] Portugal [33] China [38] Peru [41] Paraguay [42] Taiwan [43] Romania [44]	LLL	1. Need recognition [6] 2. Research endeavor [4] 3. Self-initiated learning activities [4] 4. Technical or computer skills [3] 5. Motivation [3] *N* = 20	23–44
6.	Crick et al., (2004)^a^ [45]	Effective LLL inventory	ELLI	*Education* Primary school Students [45, 47] Professionals (institutes of higher education, training organizations, private sector corporations, and staff education) [46]	US [45] UK [46, 47] Australia [46]	Learning-profile characteristics	1. Changing and learning [4] 2. Critical Curiosity [9] 3. Meaning-making [7] 4. Creativity [10] 5. Fragility and dependence [16] 6. Strategic awareness [13] 7. Learning relationships [12] *N* = 71	46; 47
7.	Wielkiewicz (2005)^a^ [48]	Wielk. Lifelong Learning scale	WLLS	*Education* Secondary school Students [48] Professionals [49] Physical education and sports teachers [50] Teacher candidates [51] *Health Care* Pediatric Nurses [52]	US [48, 49] Turkey [50–52]	Attitudes and behaviors associated with LLL	Uni-dimensional instrument, *N* = 16	49–52
8.	Moore (2009) [53]	Moore’s Survey: Attributes of a Continuous Learner	MS	*Education* Public schooling teachers [53]	US [53]	Perceptions to participating in professional development	1. Attributes of a continuous learner [11] 2. Facilitators to participation in professional development [16] 3. Negative attributes of a continuous learner [4] 4. Barriers to participation in professional development [11] 5. Characteristics of professional development [4] *N* = 45	-
9.	Coskun & Demirel (2010)^a^ [54]	LLL Tendency Scale	LLLTeS	*Education* Prospective teachers [54, 57–59, 62–65] Faculty members of educational institutions [60, 65] Primary school teachers [56, 67] Secondary school teachers [66, 68, 69] Lectures [61] Undergraduate students [61]	Turkey [54–57, 59, 60, 62–69] Abu Dhabi [58] Nigeria [61]	LLL tendencies	1. Motivation [6] 2. Perseverance [6] 3. Lack of Regulating Learning [6] 4. Lack of Curiosity [9] *N* = 27	55–69
10	Sahin et al., (2010) [70]	Scale of Key Competence for LLL	SKCLLL	*Education* Prospective teachers [70, 72, 73] University teachers [71, 74] Mathematics teacher [75]	Turkey [70–75]	Levels and views regarding LLL key competencies	1. Communicative competence at native language [4] 2. Communicative competence at a foreign language [4] 3. Mathematical basis competence at science and technology [3] 4. Digital competence [2] 5. The competence of learning to learn [2] 6. The competence of social citizenship awareness [3] 7. The competence of the sense of initiative and entrepreneurship [4] 8. The competence of cultural awareness and expression [1] *N* = 23	71–75
11.	Uzunboylu & Hursen (2011) [76]	LLL Competencies Scale	LLLCS	*Education* Prospective teachers [77] Primary school teachers [78, 80] Secondary school teachers [76, 79] Principals of primary schools [81] Physical education and sports [82]	Turkish Republic of Northern Cyprus [76, 79–81] Turkey [77, 78, 82]	LLL competencies	1. Competencies of self-management [13] 2. Competencies of learning how to learn [12] 3. Competencies of initiative and entrepreneurship (10 items) 4. Competencies of acquiring information (6 items) 5. Digital competencies [6] 6. Competencies of decision-taking [4] *N* = 51	78–82
12.	Celebi et al., (2013)^a^ [83]	LLL scale	LLLS	*Education* Teachers [83]	Turkey [83]	LLL approaches	Professional development [4] Personal development [6] Institutional development [3] *N* = 13	-
13.	Feng & Ha (2015) [84]	The LLL scale	TLLLS	*Education* Teachers from universities [84]	China [84]	LLL	1. Cognition^b^ 2. Skills^b^ 3. Affection^b^ N = not specified^b^	-
14.	Erdogan & Arsal (2016)^a^ [85]	LLL Trends Scale	LLLTS	*Education* Prospective teachers [85–87] Teachers [88–91] School administrators [91]	Turkey [85–91]	LLL trends of preservice teachers	1. Willingness to learn^b^ 2. Openness to development^b^ *N* = 17 (number of items per construct not specified)^b^	86–91
15.	Hursen (2016) [92]	LLL Attitude Scale	LLLAS	*Education* Secondary school teachers [92]	Turkey [92]	LLL attitudes	1. Reluctance to learn [7] 2. The belief in the benefit of learning activities for professional development [6] 3. Awareness of personal learning skills [6] *N* = 19	-
16.	Coffelt & Gabriel (2017) [93]	Coffelts’ instrument	CNN	*Healthcare* Occupational therapists [93]	US [93]	Continuing competence skills and practices	1. Professional development participation [9] 2. Day-to-day continuing competence practices [8] 3. Value of professional learning activities [16] 4. Importance of a professional development plan and self-reflection practice [7] 5. Continuing competence [5] 6. Concerns and promotion of professional development [5] *N* = 50	-
17.	Drewery et al., (2020) [94]	LLL Mindset	LLLM	*Accounting and finance* Professionals in accounting and finance [94]	Canada [94]	LLL mindset	1. Resilience [3] 2. Strategic thinking [4] *N* = 7	-
18	Aseffa et al., (2023) [95]	Lifelong Learning Measurement Scale	LLMS	*Education* Academic staff of higher education institutions [95]	Ethiopia [95]	Engagement in LLL	1. Learning to know [4] 2. Learning to do [4] 3. Learning to live together [5] 4. Learning to be [5] *N* = 19	-

^**a**^ Instrument included after reference checking

^**b**^ Instrument not available in English

Most instruments were developed in the US, followed by Turkey. The instruments were mostly developed for professionals working in educational contexts; however, healthcare is also represented. All instruments contained items with answering categories based on a multi-point scale. The number of items varied from 7 in the LLLM [94] to 71 in the ELLI [45]. Almost all instruments were self-administered, only the ELLI [45] is administered in person and the mode of administering the LLLM [94] was not described.

### Measurement properties

The studies developing an instrument to evaluate LLL were examined on different measurement properties, the results of the most frequently assessed are summarized and presented in Table 3. The scores are set against the maximum score of the measurement property and are not individually interpreted because the scores are divers and should be placed in their context. Therefore it is only used to compare the instruments with each other.

**Table 3:** Summary of scores on content validity, structural validity, internal consistency and construct validity per instrument measuring lifelong learning for professionals

	Instruments^a^	Content validity Population	Content validity Experts	Structural validity	Internal consistency	Construct validity	
**1**	**OCLI** [12]	33/48	24/40	9/12	9/12	22/28	
^**b**^		-	-	12/12 [14]	-		
**2**	**CLLP** [15]	-	22/40	11/12	8/12	11/28	
^**b**^		-	24/40 [17]	-	-	16/28 [16, 17]	
**3**	**SKILLS** [19]	34/48	26/40	-	-	-	
^**b**^		-	-	-	-	9/28 [20]	
**4**	**CLLPS** [21]	24/48	22/40	12/12	10/12	-	
**5**	**JSPLL** [7]	37/48	32/40	9/12	10/12	18/28	
^**b**^		-	-	11/12 [40]	12/12 [26, 29, 32, 38, 40, 43]	-	
**6**	**ELLI** [45]	30/48	12/40^c^	12/12	10/12	8/28	
^**b**^		-	-		12/12 [46, 47]	-	
**7**	**WLLS** [48]	-	-	-	7/12	9/28	
^**b**^		-	-	8/12 [43]	12/12 [50–52]	14/28 [51, 52]	
**8**	**MS** [53]	-	-	5/12	6/12	-	
**9**	**LLLTeS** [54]	33/48	28/40	11/12	9/12	-	
^**b**^		-	-	-	12/12 [57, 67]	16/28 [66, 69]	
**10**	**SKCLLL** [70]	-	25/40	9/12	10/12	-	
^**b**^		-	-	-	12/12 [74]	16/28 [74]	
**11**	**LLLCS** [76]	39/48	32/40	8/12	12/12	-	
^**b**^		-	-	12/12 [77]	-	16/28 [77]	
**12**	**LLLS** [83]	10/48	20/40	-	7/12	-	
**13**	**TLLLS** [84]	-	-	8/12	9/12	-	
**14**	**LLLTS** [85]	27/48	30/40	12/12	12/12	-	
^**b**^		-	-	-	-	16/28 [88, 91]	
**15**	**LLLAS** [92]	33/48	28/40	11/12	10/12	9/28	
**16**	**CNN** [93]	-	20/40	-	-	-	
**17**	**LLLM** [94]	-	-	10/12	10/12	11/28	
**18**	**LLMS** [95]	35/48	32/40	11/12	12/12	-	

^a^ See Table 2 for the full names of the instruments

^b^ The highest scores, if higher than the original article, on a measurement property found in any of the included validation studies

^c^ Only relevance was assessed

Note: The scores are set against the maximum score of the measurement property as defined in the scoring list

The OCLI [12] provided evidence on most measurement properties. The JSPLL [7], LLTeS [45] and LLLTS [85] provided evidence for five measurement properties, and for the other instruments evidence is provided on one to four of the measurement properties, see Additional file 3 for the results on which the scores are based [see Additional file 3]. Eleven instruments [7, 12, 15, 19, 21, 45, 49, 54, 70, 76, 85] were used or assessed for validity in one or more of the other included studies, see Additional file 4 for the scores of the measurement properties of all included studies [see Additional file 4].

### Conceptual analysis

This section provides insight into the content of the quantitative instruments, paying attention to how LLL is conceptualized, the origin of the content of instruments, the type of items and constructs of the instruments. Additional file 5 [see Additional file 5] provides a table with this information per instrument, including information on the practical use of the instrument.

### Conceptualization of LLL

All instruments vary strongly in how they define LLL. All studies used a different definition, and in most of them a link between underlying (learning) theories and their definition of LLL was not explicitly described. We identified several common elements in the definitions of lifelong learning (LLL). Nearly all define it as a continuous process throughout life, involving acquiring knowledge, skills, and attitudes for both personal and professional growth. Many definitions also highlight a range of (in)formal learning activities, and emphasize the need for LLL to adapt to a rapidly changing world, driven by technological advances. Additionally, some definitions describe key behaviors of lifelong learners, such as self-directed learning, motivation, recognizing learning needs, self-assessment, and critical thinking [see Additional file 5].

### Origin of the instruments’ content

The content of most instruments is based on an analysis of the literature and/or the input of experts and professionals/students [7, 12, 15, 45, 53, 54, 70, 76, 83–85, 92–95]. In five studies [21, 45, 48, 94, 95], a previously developed instrument served as a basis for the development of the new instrument. Two studies [12, 19] reported the use of a learning theory for the construction of the instrument; these were a theory on self-directed learning, and a theory on adult learning strategies. One study [95] used a more philosophical theory about learning and education, resulting in the four pillars of lifelong learning.

### Constructs and items used to evaluate LLL defined in the instruments

The instruments defined a wide range of constructs, with no two instruments measuring the exact same construct based on their names and included items. However, by analyzing the theoretical explanations behind these constructs, we identified eight common themes shared by two or more instruments. These themes were: learning how to learn, the belief in continuous learning for professional development, motivation to learn, professional learning activities, curiosity, digital competence, openness to change, and collaborative learning (see Table 4).

**Table 4:** Themes defined based on the constructs defined in mentioned in the instruments

Instrument^a^	How to learn	Belief importance of learning for CPD	Motivation	Learning activities	Curiosity	Digital competence	Openness to change	Learning With others
**OCLI** [12]		Yes	Yes				Yes	
**CLLP** [15]		Yes	Yes	Yes			Yes	
**SKILLS** [19]			Yes					
**CLLPS** [21]	Yes	Yes	Yes	Yes	Yes			
**JSPLL** [7]		Yes	Yes	Yes		Yes		
**ELLI** [45]	Yes				Yes			Yes
**WLLS** [48]		Yes			Yes			
**MS** [53]								
**LLLTeS** [54]	Yes		Yes		Yes			
**SKCLLL** [70]	Yes					Yes		
**LLLCS** [76]	Yes			Yes		Yes		
**LLLS** [83]			Yes					
**TLLLS** [84] ^b^								
**LLLTS** [85] ^b^								
**LLLAS** [92]	Yes	Yes	Yes					
**CNN** [93]		Yes		Yes				
**LLLM** [94]	Yes							
**LLMS** [95]	Yes			Yes				Yes

^a^ See Table 2 for the full names of the instruments

^b^ The instrument was not available in English

In addition to these construct commonalities, we also identified similarities in the items used. Most instruments included items measuring LLL attitudes (see Table 5). Other items focused on types of learning activities, both formal (e.g., reading articles, attending conferences) and informal (e.g., seeking feedback, involving others in learning). Some instruments also included items assessing learning abilities, such as goal-setting and expressing ideas in a foreign language. Lastly, items related to personal characteristics and adult learning strategies were also commonly included.

**Table 5:** Content of the items of the instruments

Instrument^a^	LLL Attitude	Learning activities	Personal characteristics	Learning skills/ strategies
**OCLI** [12]	Yes	Yes		Yes
**CLLP** [15]	Yes	Yes	Yes	Yes
**SKILLS** [19]				Yes
**CLLPS** [21]	Yes	Yes	Yes	Yes
**JSPLL** [7]	Yes	Yes		
**ELLI** [45]	Yes	Yes	Yes	Yes
**WLLS** [48]	Yes	Yes	Yes	
**MS** [53]	Yes	Yes	Yes	Yes
**LLLTeS** [54]	Yes			
**SKCLLL** [70]	Yes			Yes
**LLLCS** [76]				Yes
**LLLS** [83]	Yes	Yes		
**TLLLS** [84] ^b^				
**LLLTS** [85] ^b^				
**LLLAS** [92]	Yes			
**CNN** [93]	Yes	Yes		
**LLLM** [94]	Yes			Yes
**LLMS** [95]	Yes	Yes		Yes

^a^ See Table 2 for the full names of the instruments

^b^ The instrument was not available in English

## Discussion

This review identified 18 quantitative instruments for measuring LLL in professionals. It provides an overview of these instruments, aiding in defining and operationalizing LLL, and helping to select an appropriate tool based on its content and measurement properties for specific contexts or purposes.

### How well do the instruments measure LLL skills of professionals?

This review calls for several observations regarding the measurement properties of the instruments. The JSPLL [7] was developed specifically for health professionals, is the most widely used instrument for measuring LLL, and is assessed on validity most frequently. It provides low-risk of biased results on part of the measurement properties but not for reliability, responsiveness, and cross-cultural validity. The OCLI [12], used in nursing populations, and the LLLTeS [54], used in educational setting, provided results on most of the measurement properties. For both instruments, there is a low- risk of biased results on these measurement properties, particularly so for content validity, structural validity, and internal consistency. However, further assessment of the measurement properties is needed. Furthermore, none of the instruments studied cross cultural validity even though some instruments, such as the JSPLL [7] were used in other countries and for different populations than where originally developed for. Also responsiveness was only assessed once in all the studies [61], this may lead to uncertainty about whether the instruments are able to detect change over time and what this implicates.

Most instruments lacked a clear definition and construct presentation of LLL. Although many provided a definition, only three studies [12, 19, 95] explicitly used a learning theory in their conceptualization. While most claimed their instrument’s content was based on literature, they failed to detail the sources used. For complex, latent constructs like LLL, the accuracy of measurement depends on the construct’s clear identification and definition in the specific context. Without proper definition and operationalization, it’s unclear what is being measured, making it difficult to make valid claims about the construct [96].

### What the instruments do or do not measure

While all instruments covered a variety of constructs that operationalized LLL, there was little similarity in choice of constructs between the different instruments. This variety appears to be unrelated to the profession or population for which the instrument was designed. The JSPLL [7], CLLPS [21], and OCLI [12], are all validated for use in the healthcare setting, but each instrument covers different constructs of LLL. Some constructs, on the other hand, were covered by multiple instruments, such as motivation to learn, learning activities undertaken, curiosity, learning about your learning, digital competence, openness to change, and the belief that learning is important for professional development. These constructs might be considered as core dimensions of LLL.

Though there was some overlap in constructs across instruments, most also included unique elements. For instance, the LLMS [95] covers ‘learning to live together’ and ‘learning to be,’ which no other instrument includes. Due to the lack of consensus on the key elements of LLL in current literature, it’s impossible to assess the value of each unique domain within an LLL instrument. This highlights the importance of researchers providing clear justification for their definition and operationalization of LLL. The described implications and results of these instruments should therefore be interpreted critically.

Even though this review included a variety of types of tools to give insight into LLL skills for professionals, only quantitative questionnaires using Likert scales were found. These questionnaires have advantages in comparison to more qualitative evaluation methods, such as standardization of scores, easy to fill in and suitable for multiple participants simultaneously. However, this type of evaluation may not always be the preferred method as it requires respondents to translate their natural responses into one-dimensional responses, thus limiting their possibilities for expressing themselves [97]. It should be questioned whether this rather positivist research position in which measuring constructs is interpreted to a single score reflects the reality of multi-faceted and complex skills as LLL. It is therefore worthwhile to consider how these questionnaires can be used in a way that leaves room for qualitative input and discussion. They could for example serve to start a dialogue with professionals about their LLL development, with more room for deeper reflection on their LLL skills and discussion of the scores, which helps to better understand the results and to formulate concrete actions [98]. For example, Klug et al. developed and used an LLL interview to measure teachers’ efforts in promoting LLL amongst their students [99].

### Limitations and strengths

To capture as many tools as possible, a broad search strategy was used, leading to less than 0.5% of screened articles being included. This created a heavy workload and may have impacted screening accuracy. Reference checks identified six articles developing instruments, 33% of which were not indexed in the databases used, explaining their initial omission. Although the search was limited to English articles, the broad use of validated keywords across multiple databases minimized the risk of missing relevant instruments.

For data extraction on both the quality and content of the instruments, a robust and validated method was used, which included the use of a data extraction form that was based on the literature and piloted before final use. Although this form was based on the COSMIN [11] guidelines, the overall rating procedure developed by the COSMIN group to assess the methodological quality of the studies was not used [11] but absolute scores were used instead. Although using absolute scores may impede interpretability of the quality of the studies, it was believed that using the worst count principle, as the COSMIN prescribed, would provide too restricted results on the kind of instruments that were investigated. Our review shows that LLL is a broad concept, operationalized in different ways across the studies. While the instruments cover different aspects of LLL, none are perfect. Criticism may arise from using a tool with a positivistic stance to evaluate them, as seeking a ‘perfect’ instrument is unrealistic given the complexity of LLL.

### Implications

The results of this review support medical educators and other stakeholders involved in the training and working environment of healthcare professionals in evaluating LLL for their context, and the findings can be used as input to define, refine, and operationalize LLL. This review showed that while some LLL constructs were shared across instruments, most instruments used unique constructs, the lack of an universal definition. Therefore, stakeholders could also benefit from the definitions and operationalizations used in other domains and should not only focus on instruments developed or validated for healthcare settings.

The JSPLL [7], taking both content and methodological quality into account, seems appropriate for measuring LLL in healthcare professionals, might provide a limited view of LLL. This review yielded other instruments measuring LLL in other occupational contexts, measuring LLL in various compilations of dimensions, each emphasizing different aspects of the construct. Based on the measurement properties, the OCLI [12] and the LLLTeS [54] would appear to be promising for validation in a healthcare context. Future research could explore how LLL instruments perform across different healthcare professions and expertise levels. Furthermore, greater consensus on the definition of LLL is needed, and it is recommended that studies pay more attention to the definition and operationalization of the multi-faceted and complex construct of LLL.

## Conclusion

This review was performed to identify a tools that can be used for evaluating LLL skills among professionals in healthcare. Eighteen tools, only quantitative instruments, were indicated and their measurement properties and the content of these instruments were critically evaluated. The JSPLL [7], CLLPS [21], and OCLI [12] are all validated for use in the healthcare setting, the JSPLL [7] is assessed on validity most frequently and is the most widely used instrument for measuring LLL. The review shows the variety of how LLL has been defined, operationalized, and measured in a variety of contexts. By highlighting both the strength and limitations of the instruments, we hope to encourage their mindful use in practice.

## Electronic supplementary material

Below is the link to the electronic supplementary material.

Supplementary Material 1

## Data Availability

The data extraction form is available upon a reasonable request. Please, put in this request to M.H.M. Verkooijen (monica.verkooijen@radboudumc.nl).

## Abbreviations

LLL: Lifelong Learning
STORIES: Structured Approach to the Reporting in Healthcare Education of Evidence Synthesis
COSMIN: Consensus-Based Standards for the Selection of Health Measurement Instruments
