# Supplementary material for: How to evaluate lifelong learning skills of healthcare professionals: a systematic review on content and quality of instruments for measuring lifelong learning
Source: BMC Med Educ. 2024 Dec 5;24:1423. doi: 10.1186/s12909-024-06335-9 (PMC11622499; doi:10.1186/s12909-024-06335-9)
Supplement: Supplementary file 1 — Supplementary Material 1 [file 12909_2024_6335_MOESM1_ESM.docx]

**Additional file 1: PRISMA checklist**

| **Section and Topic** | **Item #** | **Checklist item** | **Location where item is reported** |
| --- | --- | --- | --- |
| **TITLE** | | |  |
| Title | 1 | Identify the report as a systematic review. | Line 1,2 |
| **ABSTRACT** | | |  |
| Abstract | 2 | See the PRISMA 2020 for Abstracts checklist. | Line 29-57 |
| **INTRODUCTION** | | |  |
| Rationale | 3 | Describe the rationale for the review in the context of existing knowledge. | Line 58-81 |
| Objectives | 4 | Provide an explicit statement of the objective(s) or question(s) the review addresses. | Line 83-95 |
| **METHODS** | | |  |
| Eligibility criteria | 5 | Specify the inclusion and exclusion criteria for the review and how studies were grouped for the syntheses. | Line 115-121 |
| Information sources | 6 | Specify all databases, registers, websites, organisations, reference lists and other sources searched or consulted to identify studies. Specify the date when each source was last searched or consulted. | Line 105-113 |
| Search strategy | 7 | Present the full search strategies for all databases, registers and websites, including any filters and limits used. | Additional file 2 |
| Selection process | 8 | Specify the methods used to decide whether a study met the inclusion criteria of the review, including how many reviewers screened each record and each report retrieved, whether they worked independently, and if applicable, details of automation tools used in the process. | Line 122- 133 |
| Data collection process | 9 | Specify the methods used to collect data from reports, including how many reviewers collected data from each report, whether they worked independently, any processes for obtaining or confirming data from study investigators, and if applicable, details of automation tools used in the process. | Line 135-147 |
| Data items | 10a | List and define all outcomes for which data were sought. Specify whether all results that were compatible with each outcome domain in each study were sought (e.g. for all measures, time points, analyses), and if not, the methods used to decide which results to collect. | Line 135-150 |
|  | 10b | List and define all other variables for which data were sought (e.g. participant and intervention characteristics, funding sources). Describe any assumptions made about any missing or unclear information. | Line 135-150 |
| Study risk of bias assessment | 11 | Specify the methods used to assess risk of bias in the included studies, including details of the tool(s) used, how many reviewers assessed each study and whether they worked independently, and if applicable, details of automation tools used in the process. | Line 135-150 |
| Effect measures | 12 | Specify for each outcome the effect measure(s) (e.g. risk ratio, mean difference) used in the synthesis or presentation of results. | - |
| Synthesis methods | 13a | Describe the processes used to decide which studies were eligible for each synthesis (e.g. tabulating the study intervention characteristics and comparing against the planned groups for each synthesis (item #5)). | Line 135-150 |
|  | 13b | Describe any methods required to prepare the data for presentation or synthesis, such as handling of missing summary statistics, or data conversions. | Line 135-150 |
|  | 13c | Describe any methods used to tabulate or visually display results of individual studies and syntheses. | Line 135-150 |
|  | 13d | Describe any methods used to synthesize results and provide a rationale for the choice(s). If meta-analysis was performed, describe the model(s), method(s) to identify the presence and extent of statistical heterogeneity, and software package(s) used. | Line 135-150 |
|  | 13e | Describe any methods used to explore possible causes of heterogeneity among study results (e.g. subgroup analysis, meta-regression). | - |
|  | 13f | Describe any sensitivity analyses conducted to assess robustness of the synthesized results. | - |
| Reporting bias assessment | 14 | Describe any methods used to assess risk of bias due to missing results in a synthesis (arising from reporting biases). | Line 135-150 |
| Certainty assessment | 15 | Describe any methods used to assess certainty (or confidence) in the body of evidence for an outcome. | Line 135-150 |
| **RESULTS** | | |  |
| Study selection | 16a | Describe the results of the search and selection process, from the number of records identified in the search to the number of studies included in the review, ideally using a flow diagram. | Line 153-158 and figure 1 |
|  | 16b | Cite studies that might appear to meet the inclusion criteria, but which were excluded, and explain why they were excluded. | - |
| Study characteristics | 17 | Cite each included study and present its characteristics. | Table 2 and line 164-172 |
| Risk of bias in studies | 18 | Present assessments of risk of bias for each included study. | Additional file 3 and 4, and line 178-195 |
| Results of individual studies | 19 | For all outcomes, present, for each study: (a) summary statistics for each group (where appropriate) and (b) an effect estimate and its precision (e.g. confidence/credible interval), ideally using structured tables or plots. | - |
| Results of syntheses | 20a | For each synthesis, briefly summarise the characteristics and risk of bias among contributing studies. | Additional file 3 and 4, and line 178-195 |
|  | 20b | Present results of all statistical syntheses conducted. If meta-analysis was done, present for each the summary estimate and its precision (e.g. confidence/credible interval) and measures of statistical heterogeneity. If comparing groups, describe the direction of the effect. | - |
|  | 20c | Present results of all investigations of possible causes of heterogeneity among study results. | - |
|  | 20d | Present results of all sensitivity analyses conducted to assess the robustness of the synthesized results. | - |
| Reporting biases | 21 | Present assessments of risk of bias due to missing results (arising from reporting biases) for each synthesis assessed. | - |
| Certainty of evidence | 22 | Present assessments of certainty (or confidence) in the body of evidence for each outcome assessed. | - |
| **DISCUSSION** | | |  |
| Discussion | 23a | Provide a general interpretation of the results in the context of other evidence. | Line 246 – 309 |
|  | 23b | Discuss any limitations of the evidence included in the review. | Line 311-330 |
|  | 23c | Discuss any limitations of the review processes used. | Line 311-330 |
|  | 23d | Discuss implications of the results for practice, policy, and future research. | Line 332-360 |
| **OTHER INFORMATION** | | |  |
| Registration and protocol | 24a | Provide registration information for the review, including register name and registration number, or state that the review was not registered. | Line 57, 102-103 |
|  | 24b | Indicate where the review protocol can be accessed, or state that a protocol was not prepared. | Line 102-103 |
|  | 24c | Describe and explain any amendments to information provided at registration or in the protocol. | - |
| Support | 25 | Describe sources of financial or non-financial support for the review, and the role of the funders or sponsors in the review. | Line 375 |
| Competing interests | 26 | Declare any competing interests of review authors. | Line 374 |
| Availability of data, code and other materials | 27 | Report which of the following are publicly available and where they can be found: template data collection forms; data extracted from included studies; data used for all analyses; analytic code; any other materials used in the review. | Line 371-373 |

**Additional file 2: Search strategy for each database**

|  | **ERIC** |
| --- | --- |
| **#1** | LLL/ continuous professional development  LLL/ OR  (LLL OR (Continu* ADJ2 (development OR learning))).ti,ab,id. |
| **#2** | attitude measures/ OR check lists/ OR informal assessment/ OR interviews/ OR likert scales/ OR "measures (individuals)"/ OR portfolio assessment/ OR "portfolios (background materials)"/ OR questionnaires/ OR semi structured interviews/ OR structured interviews/ OR surveys/ OR standardized tests/ OR alternative assessment/ OR "self evaluation (individuals)"/ OR graduate surveys/ OR mail surveys/ OR online surveys/ OR telephone surveys/ OR interest inventories/ OR rating scale/ OR likert scales/ OR behavior rating scale/ OR screening tests/  OR  (appraisal* OR assessment* OR check list* OR checklist* OR Conversation* OR Instrument* OR Interview* OR Inventor* OR Measurement* OR Portfolio* OR Questionnaire* OR Report OR Reports OR Scale OR scales OR Screening* OR Survey* OR tool OR tools).ti,ab,id. OR measure*.ti,id. |
| **#3** | 1 and 2 |
| **#4** | Limit 3 to (Dutch or English) |
|  | **Medline** |
| **#1** | LLL/ continuous professional development  (Continuous professional development OR continuing professional development OR LLL OR (Continu* ADJ2 (learning))).ti,ab,kf. |
| **#2** | Educational Measurement/ OR interview/ OR Self-Assessment/ OR "Surveys and Questionnaires"/ OR Checklist/ OR self report/  OR  (appraisal* OR assessment* OR check list* OR checklist* OR Conversation* OR Instrument* OR Interview* OR Inventor* OR Measurement* OR Portfolio* OR Questionnaire* OR Report OR Reports OR Scale OR scales OR Screening* OR Survey* OR tool OR tools).ti,ab,kf. OR measure*.ti,kf. |
| **#3** | 1 and 2 |
| **#4** | Limit 3 to (Dutch or English) |
|  | **Web of Science** |
| **#1** | TOPIC: (LLL/ continuous professional development  ("Continuous professional development" OR "continuing professional development" OR "LLL" OR (Continu* NEAR/1 (learning))) |
| **#2** | TOPIC: (appraisal* OR assessment* OR "check list*" OR checklist* OR Conversation* OR Instrument* OR Interview* OR Inventor* OR Measurement* OR Portfolio* OR Questionnaire* OR Report OR Reports OR Scale OR scales OR Screening* OR Survey* OR tool OR tools) |
| **#3** | TITLE: (measure*) |
| **#4** | #2 OR #3 |
| **#5** | #1 AND #4 |
| **#6** | #1 AND #4 |
| **#7** | (#1 AND #4) AND LANGUAGE: (English OR Dutch) |
|  | **Psych info** |
| **#1** | LLL/ continuous professional development  (LLL OR (Continu* ADJ2 (development OR learning))).ti,ab,id. |
| **#2** | Attitude measurement/ OR attitude measures/ OR interviews/ OR likert scales/ OR questionnaires/ OR surveys/OR mail surveys/ OR telephone surveys/ OR self-report/ OR measurement/ OR "checklist (testing)"/ OR Inventories/ OR Rating Scales/ OR interview schedules/ OR screening tests/ OR screening/ OR likert scales/  OR  (appraisal* OR assessment* OR check list* OR checklist* OR Conversation* OR Instrument* OR Interview* OR Inventor* OR Measurement* OR Portfolio* OR Questionnaire* OR Report OR Reports OR Scale OR scales OR Screening* OR Survey* OR tool OR tools).ti,ab,id. OR measure*.ti,id. |
| **#3** | 1 and 2 |
| **#4** | Limit 3 to (Dutch or English) |
|  | **Embase** |
| **#1** | LLL/ OR (Continuous professional development OR continuing professional development OR LLL OR (Continu* ADJ2 (learning))).ti,ab,kw. |
| **#2** | Measurement/ OR interview/ OR semi structured interview/ OR structured interview/ OR telephone interview/ OR Self-evaluation/ OR questionnaire/ OR open ended questionnaire/ OR structured questionnaire/OR checklist/ OR conversation/ OR self report/ OR screening/ OR screening test/ OR Rating scale/ OR attitude scale/ OR Likert scale/ OR summated rating scale/ OR (appraisal* OR assessment* OR check list* OR checklist* OR Conversation* OR Instrument* OR Interview* OR Inventor* OR Measurement* OR Portfolio* OR Questionnaire* OR Report OR Reports OR Scale OR scales OR Screening* OR Survey* OR tool OR tools).ti,ab,kw.  OR measure*.ti,kw. |
| **#3** | 1 and 2 |
| **#4** | Limit 3 to (Dutch or English) |
| **#5** | Limit 4 to conference abstracts |
| **#6** | 4 not 5 |
|  | **CINAHL** |
| **#1** | (MH “LLL”) OR TI ("LLL" OR (Continu* N1 (development OR learning))) OR AB ("LLL" OR (Continu* N1 (development OR learning))) |
| **#2** | (MH "Semi-Structured Interview") OR (MH "Structured Interview") OR (MH "Interviews") OR (MH "Self Assessment") OR (MH "Questionnaires+") OR (MH "Checklists") OR (MH "Attitude Measures") OR (MH "Conversation") OR (MH "Self Report") OR (MH "Portfolio") OR (MH "Scales") OR (MH "Surveys") OR (MH "Interview Guides") OR (MH "Structured Interview Guides") OR TI (appraisal* OR assessment* OR check list* OR checklist* OR Conversation* OR Instrument* OR Interview* OR Inventor* OR Measurement* OR Portfolio* OR Questionnaire* OR Report OR Reports OR Scale OR scales OR Screening* OR Survey* OR tool OR tools OR measure*) OR AB (appraisal* OR assessment* OR check list* OR checklist* OR Conversation* OR Instrument* OR Interview* OR Inventor* OR Measurement* OR Portfolio* OR Questionnaire* OR Report OR Reports OR Scale OR scales OR Screening* OR Survey* OR tool OR tools) |
| **#3** | 1 and 2 |
| **#4** | Limit 3 to (English) |
|  | **Sociological Abstracts** |
| **#1** | ti("LLL" OR (Continu* NEAR/1 (development OR learning))) OR ab("LLL" OR (Continu* NEAR/1 (development OR learning))) OR if("LLL" OR (Continu* NEAR/1 (development OR learning))) |
| **#2** | MAINSUBJECT.EXACT("Self Evaluation") OR MAINSUBJECT.EXACT("Conversation") OR MAINSUBJECT.EXACT("Measures (Instruments)") OR MAINSUBJECT.EXACT("Scales") OR MAINSUBJECT.EXACT("Tests") OR MAINSUBJECT.EXACT("Attitude Measures") OR MAINSUBJECT.EXACT("Questionnaires") OR MAINSUBJECT.EXACT("Surveys") OR MAINSUBJECT.EXACT("Interviews") OR MAINSUBJECT.EXACT("Telephone Surveys") OR MAINSUBJECT.EXACT("Mail Surveys") OR MAINSUBJECT.EXACT("Interview Schedules") OR ti(appraisal* OR assessment* OR check list* OR checklist* OR Conversation* OR Instrument* OR Interview* OR Inventor* OR Measurement* OR Portfolio* OR Questionnaire* OR Report OR Reports OR Scale OR scales OR Screening* OR Survey* OR tool OR tools OR measure*) OR ab(appraisal* OR assessment* OR check list* OR checklist* OR Conversation* OR Instrument* OR Interview* OR Inventor* OR Measurement* OR Portfolio* OR Questionnaire* OR Report OR Reports OR Scale OR scales OR Screening* OR Survey* OR tool OR tools) OR if(appraisal* OR assessment* OR check list* OR checklist* OR Conversation* OR Instrument* OR Interview* OR Inventor* OR Measurement* OR Portfolio* OR Questionnaire* OR Report OR Reports OR Scale OR scales OR Screening* OR Survey* OR tool OR tools OR measure*) |
| **#3** | 1 and 2 |
| **#4** | Limit 3 to (Dutch and English) |

**Additional file 3: Results on measurement properties of each included article**

| **Studies** | **Instrument** | **Structural validity** | **Internal consistency** | **Cross-cultural**  **validity** | **Reliability** | **Measure-ment error** | **Construct validity** | **Respons-**  **iveness** |
| --- | --- | --- | --- | --- | --- | --- | --- | --- |
| **1(12)*** | Oddi Continuing Learning Inventory | Factor analysis yielded five factors accounting for 44.5% of the total variance. | Analysis of the remaining 24 items yielded an internal consistency of .875 | - | Test-retest reliability was .893 | - | Scores on the OCLI correlated positively (r= .363, p=.004) with scores on the LAS. Scores also correlated positively with scores on numerous subscales of the ACL. These positive correlations suggest convergent validity of the OCLI. A measure of discriminant validity was provided when scores on the OCLI failed to correlate significantly (r - .040, p= .754) with scores on the Shipley. | - |
| **1(13)** | OCLI | - | Cronbach’s α of .90 | - | - | - | There was no significant correlation between OCLI scores and voluntary attendance at continuing education. The OCLI scores were significantly positively correlated to the scores on the Inquiry, Performance, and Self-instruction subscales of the JAS. There was no significant correlation between the OCLI scores and scores on the Group Instruction subscale of the JAS. | - |
| **1(14)** | OCLI | Using the Gorsuch three-sample approach, it was indicated that the three factors derived from the Six (1987) data set match those reported by Oddi (1984). | Cronbach’s α for Six, Oddi, and Landers were .77, .85, and .77, respectively. | - | - | - | - | - |
| **2(15)*** | Characteristics of Lifelong Learners in the Professions | Confirmatory factor analysis yielded 6 Factors (Eigenvalue >1), accounting for 44.7% of the total variance after rotation. | Cronbach’s α of 0.91 | - | - | - | Convergent validity: to predict the single dependent variable, the average number of hours per month spent in learning activities, which served as the measure of LLL Educability and Future Orientation emerged as statistically significant predictors of time spent in learning activities over the previous five years. | - |
| **2(16)** | CLLP | - | - | - | - | - | Convergent validity: The bipolar factor, Organized, was the only variable associated with time spent in learning activities only in the past year. The three Adjective Check List scales comprising this factor were Order, Endurance, and Change. | - |
| **2(17)** | CLLP | Exploratory factor analysis yielded seven Factors, accounting for 45.6% of the total variance before rotation. | - | - | - | - | Convergent validity: The dependent variable was the amount of time spent in learning activities over the previous year. As previously discussed, the dependent variable consisted of an averaged score based on participants' reported number of professional learning activities checked on the activity survey list of 42 educational activities. Self-Motivated Learning, External Motivation, and Educational Level (dimensions of the CLLP) were found to significantly predict time spent in learning activities over the previous year. | - |
| **2(18)** | CLLP | Exploratory factor analysis yielded four Factors (Eigenvalue >1), accounting for 42.5% of the total variance after rotation. | - | - | - | - | Known-group validity:  1) Self-motivated learning: A significant difference was found: participants who were self-motivated scored higher on multiple aspects of the CLLP, for example: More interested in reading and evaluate their own learning. 2) External motivation: External motivation was predictive of the amount of time spent in learning activities. | - |
| **3(19)** | Self-Kowledge Inventory of LLL Strategies | - | - | - | Spearman-Brown = 0.84; Guttmansplit-half = 0.84 | - | - | - |
| **3(20)** | SKILLS | - | Cronbach’s α of .71 | - | Spearman-Brown = 0.83 | - | Known-group validity: 1) Associate degree/baccalaureate program: The ADN from the BSN students can be discriminated on Reward/Enjoyment, Attention, using external aids, conditional acceptance, and using human resources. | - |
| **4(21)*** | Characteristics of Lifelong Learners in the Professions Scale | Exploratory factor analysis yielded 6 Factors (Eigenvalue >1), accounting for 52.1% of the total variance after rotation. | Cronbach’s α of 0.92 | - | - | - | - | - |
| **4(22)** | CLLPS | - | - | - | - | - | - | - |
| **5(7)*** | Jefferson Scale of Physician LLL | Exploratory factor analysis yielded five Factors (Eigenvalue >1), accounting for 60% of the total variance before rotation. | Cronbach’s α per component:  Need recognition = 0.99  Research endeavor = 0.82  Self-initiation = 0.75  Technical skills = 0.74  Personal motivation = 0.65 | - | - | - | Convergent validity:  Statistically significant correlation with a 10-point global scale of LLL (r > 0.20, p<0.05) | - |
| **5(23)** | JSPLL | - | Cronbach’s α of .85 | - | - | - | - | - |
| **5(24)** | JSPLL | - | Cronbach’s α of .848 | - | - | - | - | - |
| **5(25)** | JSPLL (14 item version) | - | - | - | - | - | Known-group validity:  1) Gender: No significant difference was found, except for research endeavor where men outscored women (effect size = 0.50).  2) Professional activities: Physicians who published papers, presented research findings at professional meetings or collaborated in the conduct of research obtained significantly higher mean scores on each of the five factors compared with those who were not involved in these types of activities.  Known-group validity: 1) Years of experience: A significant relation between nurses' orientation towards LLL and years of clinical experience (r = -0.144, p<0.05). 2) Age: A significant relation between nurses' orientation towards LLL and age (r = -0.192, p<0.05). 3)  Level of education: A significant relation between nurses' orientation towards LLL and level of education (r = 0.211, p<0.01). | - |
| **5(26)** | JSPLL | - | Cronbach’s α of >.70 | - | - | - | Known-group validity: 1) Discipline: Physicians showed higher abilities toward LLL than nurses (t = 3.92, p <0.001). A significant relation between nurses' orientation towards LLL and years of clinical experience (r = -0.144, p<0.05). 2) Gender: Women showed higher scores on subscale somatization than men (p<0.01). | - |
| **5(27)** | JSPLL | - | Cronbach’s α of 0.88, and per subscales ranging from 0.72 to 0.82 | - | - | - | Convergent validity: The JSPLL demonstrated convergent validity with the five-item measure of involvement in professional activities that assessed ongoing engagement in learning, r=.52. In addition, the JSPLL demonstrated moderate convergent validity with involvement in informal CE (r= .38) and formal CE (r = .24). Likewise, psychologists who invested more in LLL also reported higher levels of professional competence, as measured by the Professional Competence Scale (r = .61). | - |
| **5(28)** | JSPLL | - | - | - | - | - | - | - |
| **5(29)** | JSPLL | Four factors with eigenvalues greater than 1 accounted for 60% of the total variance. Each factor can be considered as a subscale of the JSPLL. | Cronbach’s α per component:  professional learning beliefs and motivation = 0.79. scholarly activities = 0.89. Attention to learning opportunities = 0.74. technical skills in seeking information =0.82. | - | Test-retest reliability was 0.91 (Pearson correlation) | **-** | 26 additional items (1 global indicator of LLL, 13 supplementary, and 12 checklist items) to assess validity. Subscales are consistent with the competencies of self-directed learning such as skills for information retrieval, motivation and self-initiation, attention to learning opportunities and scholarly activities, and identification of learning needs, thus providing support for the construct validity of the scale. | - |
| **5(30)** | JSPLL (student version) | - | Cronbach’s α of the subscales ranged from 0.52 to 0.96 | - | - | - | significantly. correlated with the EBM Scale (r=0.4, p =0.035), InfoUse1 (r=0.41, p =0.032), and InfoUse2 (r=0.49, p =0.009). However, no significant correlation was found between the JSPLL Scale and self-efficacy. As to the EBM Scale, it was not significantly correlated with InfoUse1, InfoUse2, and self-efficacy. A strong association was observed between InfoUse1 and InfoUse2 (r=0.71, p <0.001), while self-efficacy was moderately correlated with InfoUse1 and InfoUse2. | - |
| **5(31)** | JSPLL (14 item version) | - | Cronbach’s α of .81 | - | - | - | The main effects of psychological need satisfaction and involvement in clinical teaching were determined to be significant in physician LLL (P = .004 and P = .037, respectively). | - |
| **5(32)** | JSPLL | Exploratory factor analysis yielded four Factors. | Cronbach’s α per component: Professional learning beliefs and motivation = 0.87, scholarly activities = 0.87, Attention to learning opportunities = 0.82, technical skills in information seeking = 0.89 | - | - | - | - | - |
| **5(33)** | JSPLL | Exploratory factor analysis was performed to assess the four Factors. | Cronbach’s α of .842 | - | - | - | - | - |
| **5(34)** | JSPLL | - | - | - | - | - | JSPLL scores were significantly correlated with AMS-RAM scores (r = 0.39; P < .001). For the IM domain, JSPLL was significantly positively associated with IM to know (r = 0.46; P < .001), to accomplish things (r = 0.35; P < .001), and to experience stimulation (r = 0.23; P = .021). For the EM domain, only EM through external regulation was significantly negatively correlated with JSPLL scores (r = −0.20; P = .047). Amotivation was not significantly associated with JSPLL scores. Analysis of JSPLL factor scores also showed significant correlations with AMS subdomains. Additionally, CSS residents had significantly higher JSPLL scores than non-CSS residents. | - |
| **5(35)** | JSPLL | - | - | - | - | - | - | - |
| **5(36)** | JSPLL | - | - | - | - | - | Known-group validity:  Career tracks: No significant difference was found between academic career tracks. | - |
| **5(37)** | JSPLL | - | - | - | - | - | - | - |
| **5(38)** | JSPLL | Three factors with eigenvalues greater than 1 accounted for 60.64% of the total variance. | Cronbach’s α per factor: learning beliefs and motivation= 0.837; attention to learning opportunities=0.754; technical skills in seeking information= 0.663. | - | - | - | - | - |
| **5(39)** | JSPLL (Revised) | Three factors  accounted for 52% of the variance among full-time clinicians and 54% of the variance among academic clinicians. | Cronbach’s α was equally large for both groups (0.85 and 0.86, respectively). | - | Test-retest reliability coefficients (approximately four months between testing) were at an acceptable range for psychological testing (0.72 and 0.77, respectively | - | Other instruments: Scores on the JSPLL were correlated with responses to 13 indicators of learning motivation. All the correlations were statistically significant for both groups.  Between groups: We compared the scores on the JSPLL for physicians classified in the top 25%, middle 50%, and bottom 25% class rank. The mean scores on the JSPLL increased with increasing levels of class rank. | - |
| **5(40)** | JSPTLL revised for physical therapists | Factor analysis yielded three factors accounting for 55.3% of the total variance. | Cronbach’s α of 0.95, and per component:  Attention to learning opportunities = 0.86  Learning beliefs and motivation = 0.87  Skills in seeking information = 0.81 | - | Test-retest reliability was .89 | - | Convergent validity: A medium correlation exists between the JSPTLL and the level of career satisfaction (rs = 0.32, p<00.0002) A small correlation exists between commitment to lifelong learning and levels of career satisfaction (r=0.27). | - |
| **5(41)** | JSPLL | - | Cronbach’s α of 0.83 and per component >0.80 | - | - | - | - | - |
| **5(42)** | JSPTLL | - | Cronbach’s α of 0.70 | - | - | - | Convergent validity: Having higher lifelong learning abilities (p < 0.001) appeared to be predictors of greater somatization in healthcare professionals. Having lower development in lifelong learning abilities (p = 0.01) appeared to be predictors of greater work alienation. | - |
| **5(43)** | JSPLL revised | Factor analysis yielded two factors accounting for 60.5% of the total variance. | Cronbach’s α per component:  Learning beliefs and motivation = 0.89  Attention to learning opportunities = 0.83 | - | - | - | Convergent validity: Orientation toward lifelong learning was positively related to job demands (r=0.19, P<0.01), job control (r=0.40, P<0.001), social support from supervisors (r=0.30, P<0.001) and social support from colleagues (r=0.28, P<0.001). | - |
| **5(44)** | JSPLL revised | - | Cronbach’s α of 0.91 | - | - | - | Convergent validity: Table 2 shows the results more elaborate. Medical. Lifelong learning M45.66 (SD0.31) −0.13 * 0.29 ** −0.11 * 0.13 **  Nonmedical. Lifelong learning M45.03 (SD0.52) 0.10 0.37 ** 0.05 0.07 | - |
| **6(45)*** | Effective LLL inventory | Confirmatory factor analysis yielded seven Factors; total variance explained = 35.3%.  Exploratory factor analysis yielded 16 Factors (Eigenvalue >1), accounting for 51.1% of the total variance. | Cronbach’s α per component:  Growth orientation = 0.69  Curiosity, energy = 0.71  Meaning-making = 0.62  Creativity = 0.68  Dependence = 0.70  Learning relationships = 0.68  Strategic awareness = 0.50 | - | - | - | Known-group validity:  1) Gender: A significant difference was found: girls outscored on dependence and fragility, creativity and learning relationships; boys scored higher on strategic awareness and critical curiosity.  2) Age: p<. 05 level in scores for the three age groups (7-11ys, 11-14ys, 14-18ys) on five dimensions. These were: growth orientation F=10.78 p=.000; meaning-making F=5.48, p=004; creativity F=15.02, p=000, critical curiosity F=9.328 p=000, and learning relationships F=15.161, p=.000. | - |
| **6(46)** | ELLI | Exploratory factor analysis yielded 14 Factors (Eigenvalue >1), accounting for 49.1% of the total variance after rotation. | Cronbach’s α per component:  Changing and learning = 0.75 Critical curiosity = 0.76 Meaning making = 0.75 Creativity = 0.79 Strategic awareness = 0.85 Learning relationships = 0.72 Fragility and dependence = 0.82 | - | - | - | - | - |
| **6(47)** | ELLI | Principal component analyses yielded 18 factors, accounting for 50.73% of the total variance after Oblimin rotation and Kaiser normalisation. | Cronbach’s α per scale  Changing and learning = .69 Critical curiosity = .72 Meaning making = .65 Creativity = .80 Learning relationships = .74 Strategic awareness = .82 Fragility and dependence = .84 | - | - | - | - | - |
| **7(48)*** | WielkLLS | - | Cronbach’s α of 0.75 | - | - | - | Convergent validity: The relation of the LLL to Systemic Thinking was also fairly substantial (r= -.28). Students who think more systemically tend to have higher scores on the LLL scale. Higher scores on the LLL scale were also associated with higher scores on Astin's Social Activist type. Finally, the LLL scale made a significant contribution to predicting GPA. | - |
| **7(49)** | WLLS | Exploratory factor analysis yielded one Factor. | Cronbach’s α of 0.71 | - | - | - | Convergent validity: As predicted, the WielkLLS was positively associated with college GPA, negatively associated with alcohol use, positively associated with Systemic Thinking (the negative r reported in Table 3 reflects scale construction), and positively associated with the Intellect/ Imagination scale of the mini-IPIP. A weaker tendency toward hierarchical thinking was associated with a higher score on the WielkLLS and higher GPA. | - |
| **7(50)** | WLLS | Single factor solution showed 52.7 | Cronbach’s α of .94 | - | - | - | - | - |
| **7(51)** | WLLS | - | Cronbach’s α of 0.83 | - | - | - | Convergent validity: Constructivist conception was found to be positively related to (...) lifelong learning (r= .34, p<.01). Self-efficacy in organizing out-of-school trips was found to be positively related to lifelong learning (r= .35, p<.01). There was no significant relationship between lifelong learning and traditional conception and constructivist conception. | - |
| **7(52)** | WLLS | - | Cronbach’s α of 0.93 | - | - | - | Convergent validity: LLL significantly predicted their score on the “using resources to collect information and listening carefully” subdimension of the self‐confidence with clinical decision‐making  scale (β = 0.289). The “using information to see the big picture” subdimension of the self‐ confidence with clinical decision‐making scale was found to be significantly influenced by LLL (β = 0.647), as well as “knowing and acting” (β = 0.545). LLL did not significantly predict the score on the subdimensions of the anxiety with clinical decision‐making scale (β = 0.032, β = 0.026, β = 0.038. | - |
| **8(53)*** | Moore’s Survey | Factor analysis yielded fourteen Factors. | Cronbach’s α of .525 | - | - | - | - | - |
| **9(54)*** | LLL tendency scale | Exploratory factor analysis yielded four Factors, accounting for19.6% of the total variance after rotation. | Cronbach’s α of 0.89 | - | - | - | - | - |
| **9(55)** | LLLTeS | - | Cronbach’s α of .91 | - | - | - | - | - |
| **9(56)** | LLLTeS | - | Cronbach’s α of 0.92 |  |  | - | - | - |
| **9(57)** | LLLTeS | - | Cronbach’s α of 0.92, and per component:  Motivation = 0.85 Persistence = 0.88 Lack of Learning in Regulation = 0.83 Lack of Curiosity = 0.89 | - | - | - |  | - |
| **9(58)** | LLLTeS | - | - | - | - | - | - | - |
| **9(59)** | LLLTeS | - | Cronbach’s α of .72 | - | - | - | - | - |
| **9(60)** | LLLTeS | - | - | - | - | - | - | - |
| **9(61)** | LLLTeS | Confirmatory factor analysis yielded four Factors. | Cronbach’s α per component:  Motivation = 0.89-0.92 Perseverance = 0.88-0.91 Self-regulation = 0.86-0.91 Curiosity = 0.87-0.90 | - | - | - | - | Time and group interaction at T1 versus T2 (F(1, 60) = 228.929,p< .001,ŋ2p= .589), and T1 versus T3 (F(1, 60) = 229.729,p< .001,ŋ2p= .589) in the within-subjects effects test reveals significant effects, respectively. The test of between-subjects effects for the interaction groups at T1 versus T2 (F(1, 60) = 43.813,p<.001,ŋ2p= .215), and T1 versus T3 (F(1, 60) =67.934,p<.001,ŋ2p= .298) are statistically significant, respectively. |
| **9(62)** | LLLTeS | - | Cronbach’s α of .92 | - | - | - |  | - |
| **9(63)** | LLLTeS | - | Cronbach’s α of .91 | - | - | - | The whole relation between learning-oriented motivation and teacher self-efficacy of candidate teachers (direct relation estimates=.26) is provided through LLL tendencies (indirect relation estimates=.27), and LLL tendencies is the full mediator variable for this theoretical model. |  |
| **9(64)** | LLLTeS | - | - | - | - | - | - | - |
| **9(65)** | LLLTeS | - | - | - | - | - | - | - |
| **9(66)** | LLLTeS | - | Cronbach’s α of .93 | - | - | - | Convergent validity: A moderate, positive and signifcant relationship between technology integrating self-efficacy and lifelong learning tendencies was found (r = 0.412, p <.01.), asweel in english teachers’ special feld competencies and lifelong learning tendencies (r = 0.349, p <.01.). | - |
| **9(67)** | LLLTeS | Factor analysis yielded four factors | Cronbach’s α of 0.90, and per component: Motivation = 0.72  Perseverance = 0.87  Lack of Regulating Learning = 0.75  Lack of Curiosity = 0.92 | - | - | - | - | - |
| **9(68)** | LLLTeS | - | Cronbach’s α of 0.80, and per (unknown) component: 0.87; 0.80; 0.78; 0.86 | - | - | - | Convergent validity: The independent variable, LLL tendency, has the highest correlation with the ‘years of job experience’ variable (r = 0.399 p < 0.01) and has the lowest correlation with the ‘type of school’ variable (r = −0.014; p < .05) | - |
| **9(69)** | LLLTeS | -- | Cronbach’s α of 0.90 | - | - | - | Convergent validity: A positive and low-level relationship between perceived innovation management competencies of administrators and teachers' lifelong learning tendencies (r=.194; p<.01); there is a positive and moderate significant relationship is found among LLT of teachers and their individual innovativeness levels (r=.414; p<.01). | - |
| **10(70)*** | Scale of Key Competence for LLL | The factor analysis yielded eight factors. | Cronbach’s α of .75 | - | - | - | - | - |
| **10(71)** | SKCLLL | - | Cronbach’s α of .88 | - | - | - | - | - |
| **10(72)** | SKCLLL |  | Cronbach’s α of 0.75 | - | - | - | - | - |
| **10(73)** | SKCLLL | - | Cronbach’s α of .89 | - | - | - | - | - |
| **10(74)** | SKCLLL | - | Cronbach’s α per component:  Native language communication = 0.778  Foreign language communication = 0.940 Basic mathematical competency in science and technology = .670 Digital = 0.797 Learning to learn = 0.840 Social citizenship = .846 Initiative and entrepreneurship comprehension = 0.849 | - | - | - | Occupational motivation is not a significant predictor of LLL competencies and its subdimensions of teachers (F = 0.243, p>0.5). | - |
| **10(75)** | SKCLLL | - | Cronbach’s α of 0.77 | - | - | - | - | - |
| **11(76)*** | LLL competence scale | Exploratory factor analysis yielded six Factors (Eigenvalue >1), accounting for 59% of the total variance before rotation. | Cronbach’s α per component:  Self-management competencies = 0.93  Competencies of learning how to learn = 0.91  Competencies of Initiative and entrepreneurship = 0.89  Competencies on acquiring information = 0.83  Digital Competencies = 0.85  Competencies of decision-taking = 0.75 | - | - | - | - | - |
| **11(77)** | LLLCS | Confirmatory factor analysis yielded six Factors. | - | - | - | - | This study revealed that a statistically significant correlation between self-competency beliefs (covariate variable set) and LLL competency beliefs (dependent variable set) existed and explained a total of 44% of the covariance between the two belief sets. | - |
| **11(78)** | LLLCS | Principal component analyses yielded six Factors (Eigenvalue 3,52), explaining  70.37% of the total variance. | Cronbach’s α  Self-management = 0.94 Learning how to learn = 0.93 Initiative and entrepreneurship = 0.94 Information acquisition = 0.82 Decision making = 0.89 Digital competencies = 0.86 | - | - | - | - | - |
| **11(79)** | LLLCS | - | - | - | - | - | Convergent validity: Statistically significant correlation between the teachers’ attitudes and perceptions of competence regarding LLL (r = 0.424, r2 = 0.179, p<0.01) | - |
| **11(80)** | LLLCS | - | Cronbach’s α per component: self-direction = 0.965; Learning to learn = 0.972, Sense of initiative and entrepreneurship = 0.994; Obtaining knowledge = 0.897, Digital = 0.736, Decision-making = 0.750 | - | - | - | - | - |
| **11(81)** | LLLCS | - | Cronbach’s α of 0.97, and per component:  Self-direction competence = 0.960 Learning to learn competence = 0.969 Sense of initiative and entrepreneurship competence = 0.992 Obtaining knowledge competence = 0.894 Digital competence = 0.739 Decision-making competence = 0.756 | - | - | - | - | - |
| **11(82)** | LLLCS | Factor analysis yielded six factors | Cronbach’s α of 0.95, and per component:  Competencies of self-management = 0.91  Competencies of learning how to learn= 0.91  Competencies of initiative and entrepreneurship = 0.89  Competencies of acquiring information = 0.83  Digital competencies = 0.85  Competencies of decision-taking = 0.85 | - | - | - | - | - |
| **12(83)*** | LLL Scale | - | Cronbach’s α of 0.823 | - | - | - | - | - |
| **13(84)*** | LLLS | Exploratory factor analysis yielded three Factors, total variance explained = 76.873%. | Cronbach’s α per component:  Cognition = 0.85 Skills = 0.82 Affection = 0.89 | - | - | - | - | - |
| **14(85)*** | LLL Trends Scale | Confirmatory factor analysis yielded two Factors (Eigenvalue >1), total variance explained = 43.44%. | Cronbach’s α of 0.86, and per component:  Willingness to learn = 0.82 Willingness to improvement = 0.82 | - | ω-value of 0.89 and test-retest reliability of 0.76 | - | - | - |
| **14(86)** | LLLTS | - | Cronbach’s α of 0.86  Cronbach’s α of 0.86, and per component:  Openness to development = 0.82 Willingness to learn = 0.82 | - | - | - | There was a statistically significant relation between LLL trends and all factors of OISS. LLL trends were found to be medium negatively correlated to disorientation (r=‐.521; p<0.01) and medium positively correlated to evaluation (r=.408; p<0.01), purposeful thinking (r=.431; p<0.01), trial & error (r=.387; p<0.01), select main ideas (r=.419; p<0.01), control (r=.336; p<0.01), and problem‐solving (r=.548; p<0.01) factors of OISS. The highest level of relation was found between LLL trends and problem‐solving factor of OISS. | - |
| **14(87)** | LLLTS | - | - | - | - | - | - | - |
| **14(88)** | LLLTS | Factor analysis yielded two factors accounting for 34.4% of the total variance. | Cronbach’s α of .86 | - | Test-retest reliability was .76 | - | Convergent validity: A moderately significantly positive correlation was found between digital literacy and lifelong learning tendencies (r = .55, p < .01). | - |
| **14(89)** | LLLTS | - | Cronbach’s α of .86 | - | - | - | Convergent validity: Positive correlation was significantly found between the scores of lifelong learning tendencies and digital literacy levels of teachers (r =,173, p<.05). | - |
| **14(90)** | LLLTS | Factor analysis yielded two factors | Cronbach’s α of 0.90 | - | - | Lifelong learning S.E. = 0.064 | Convergent validity: digital literacy predicts lifelong learning disposition positively and statistically significantly (β0=.663; β1=.485; p<.001). Digital literacy explains 44% of the changes in lifelong learning diposition. Lifelong learning disposition predicts creative thinking disposition in a positive and statistically significant way (β0=.595; β1=.565; p<.001). | - |
| **14(91)** | LLLTS | - | Cronbach’s α of 0.88 | - | - | - | Convergent validity: The correlation coefficient between lifelong learning tendencies and innovative behavior level was found as 0.534, p<0.00. This value indicates a positive, moderate relation. Correlation coefficient between lifelong learning tendencies and entrepreneurial behavior levels among the examined relations was found as 0.377. | - |
| **15(92)*** | LLL Attitude Scale | Factor analysis yielded three Factors (Eigenvalue >1),  accounting for 54% of the total variance. | Cronbach’s α per component:  Reluctance to learn = 0.84  Belief in the benefit of learning activities for professional development = 0.85  Awareness of personal learning skills = 0.78 | - | - | - | Known-group validity:  1) Work experiences: No significant difference was found except between the teachers’ work experience and their awareness of personal learning skills (F (3; 296) = 3.721, p<0.05). | - |
| **16(93)** | Coffelts’ no name | - | - | - | - | - | - | - |
| **17(94)*** | LLL Mindset | Two factors were found, explaining 40.7 percent of the variance. | Cronbach’s α of .70 | - | - | - | Convergent validity: Higher scores on the LLLM questionnaire were associated with a greater number of promotions, greater job satisfaction, greater work engagement, and more job-related self-efficacy. | - |
| **18***  **(95)** | LLMS | Factor analysis yielded four factors accounting for 56.0% of the total variance. | Cronbach’s α of 0.86, and per component:  Learning to know = 0.79; Learning to do = 0.81; Learning to live together = 0.87; Learning to be = 0.86 | - | - | - | - | - |

**Additional file 4: Scores on all measurement properties in all included studies**

| **Studies** | **Instrument** | **Country** | **Target population** | **Content validity  *Target population*** | **Content validity**  ***Experts***  ***Expert*** | **Structural validity** | **Internal consistency** | **Cross-cultural validity** | **Reliability** | **Measurement error** | **Construct validity** | **Responsiveness** |
| --- | --- | --- | --- | --- | --- | --- | --- | --- | --- | --- | --- | --- |
| **1* (12)** | Oddi Continuing Learning Inventory | US | Education- Graduate students in Law, Nursing and Adult Education | 33/48 | 24/40 | 9/12 | 9/12 | - | 4/8 | - | 22/28 | - |
| **1 (13)** | OCLI | US | Registered nurses | - | - | - | 7/12 | - | - | - | 11/28 | - |
| **1 (14)** | OCLI | US | Education- Graduate students in Law, Nursing and Adult Education | - | - | 12/12 | 9/12 | - | - | - | - | - |
| **2* (15)** | Characteristics of Lifelong Learners in the Professions | US | Human Services professionals | - | 22/40 | 11/12 | 8/12 | - | - | - | 11/28 | - |
| **2 (16)** | CLLP | US | Human Service professionals | - | - | - | - | - | - | - | 16/28 | - |
| **2 (17)** | CLLP | US | Human Service professionals | - | 24/40 | 10/12 | - | - | - | - | 16/28 | - |
| **2 (18)** | CLLP | US | Educators | - | - | 11/12 | - | - | - | - | 12/28 | - |
| **3*(19)** | Self-Knowledge Inventory of LLL  Strategies | US | Diverse - Including educators, students,  clerical workers, farmers, blue-collar workers, and homemakers | 34/48 | 26/40 | - | - | - | 7/8 | - | - | - |
| **3(20)** | SKILLS | US | Nursing students | - | 20/40 | - | 7/12 | - | 3/8 | - | 9/28 | - |
| **4* (21)** | Characteristics of Lifelong Learners in the Professions Scale | US | Healthcare- Pharmacists | 24/48 | 22/40 | 12/12 | 10/12 | - | - | - | - | - |
| **4 (22)** | CLLPS | US | Healthcare- Pharmacists | - | - | - | - | - | - | - | - | - |
| **5* (7)** | Jefferson Scale of Physician LLL | US | Healthcare -Physicians | 37/48 | 32/40 | 9/12 | 10/12 | - | - | - | 18/28 | - |
| **5 (23)** | JSPLL | The Netherlands | Healthcare -Physicians | - | - | - | 9/12 | - | - | - | - | - |
| **5 (24)** | JSPLL | The Netherlands | Healthcare -Physicians | - | 14/40 | 9/12 | 9/12 | - | - | - | - | - |
| **5(25)** | JSPLL (14-item version) | Uganda | Nurses | 17/48 | - | - | - | - | - | - | 10/28 | - |
| **5 (26)** | JSPLL | Latin America | Healthcare -Physicians & Nurses | - | - | - | 12/12 | - | - | - | 11/28 | - |
| **5 (27)** | JSPLL | US | Psychologists | - | - | - | 10/12 | - | - | - | 16/28 | - |
| **5(28)** | JSPLL | US | Healthcare - Residents | **-** | **-** | **-** | **-** | **-** | **-** | **-** | **-** | **-** |
| **5 (29)** | JSPLL | US | Healthcare -Physicians | - | - | 11/12 | 12/12 | - | 5/8 | - | 15/28 | - |
| **5 (30)** | JSPLL – (student version) | US | Healthcare - Residents | - | - | - | 7/12 | - | - | - | 13/28 | - |
| **5 (31)** | JSPLL (14 item version) | Canada | Healthcare -Physicians | - | - | - | 9/12 | - | - | - | 14/28 | - |
| **5 (32)** | JSPLL | Iran | Medical Science staff | 36/48 | 28/40 | 10/12 | 12/12 | - | - | - |  | - |
| **5 (33)** | JSPLL | Portugal | Healthcare -Physicians | - | - | 6/12 | 9/12 | - | - | - | - | - |
| **5 (34)** | JSPLL | US | Healthcare | - | - | - | - | - | - | - | 25/28 | - |
| **5 (35)** | JSPLL | Canada | Healthcare -Physicians | - | - | - | - | - | - | - | - | - |
| **5 (36)** | JSPLL | US | Healthcare - Residents | - | - | - | - | - | - | - | 21/28 | - |
| **5 (37)** | JSPLL | US | Healthcare -Physicians | - | - | - | - | - | - | - | - | - |
| **5 (38)** | JSPLL | China | Healthcare -Physicians | - | - | 11/12 | 12/12 | - | - | - | - | - |
| **5 (39)** | JSPLL (Revised) | US | Healthcare -Physicians | 32/48 | - | 11/12 | 9/12 | - | 6/8 | - | 26/28 | - |
| **5 (40)** | JSPLL | USA | Health Care – physical therapists | 30/48 | 22/40 | 11/12 | 12/12 | - | 6/8 | - | 10/28 | - |
| **5 (41)** | JSPLL | Peru | Health Care - professionals | - | - | - | 9/12 | - | - | - | - | - |
| **5 (42)** | JSPLL | Paraguay | Health Care – physicians and nurses | - | - | - | 9/12 | - | - | - | 16/28 | - |
| **5 (43)** | JSPLL | Taiwan | Health Care – physicians | - | - | 9/12 | 12/12 | - | - | - | 16/28 | - |
| **5 (44)** | JSPLL | Romania | Health Care - specialists | - | - | - | 9/12 | - | - | - | 16/28 | - |
| **6* (45)** | Effective LLL inventory | UK | Education- Primary school Students | 30/48 | 12/40** | 12/12 | 10/12 | - | - | - | 8/28 | - |
| **6 (46)** | ELLI | UK | Professionals (higher education, training organizations, private sector corporations, staff from primary, secondary and other) | - | - | 11/12 | 12/12 | - | - | - | - | - |
| **6 (47)** | ELLI | UK | Education- Primary school Students | - | - | 11/12 | 12/12 | - | - | - | - | - |
| **7*(48)** | WielkLLS | US | Education- Secondary school Students | - | - | - | 7/12 | - | - | - | 9/28 | - |
| **7 (49)** | WLLS | US | Education - Professionals | - | - | 8/12 | 10/12 | - | - |  | 13/28 | - |
| **7 (50)** | WLLS | Turkey | Education –  Sport teacher | - | - | 6/12 | 12/12 | - | - | - | - | - |
| **7 (51)** | WLLS | Turkey | Education – teacher and candidates | - | - | - | 12/12 | - | - | - | 14/28 | - |
| **7 (52)** | WLLS | Turkey | Health Care – pediatric nurses | - | - | - | 12/12 | - | - | - | 14/28 | - |
| **8* (53)** | Moore’s Survey | US | Education- Public schooling teachers | - | - | 5/12 | 6/12 | - | - | - | - | - |
| **9* (54)** | LLL tendency scale | Turkey | Education- Prospective teachers | 33/48 | 28/40 | 11/12 | 9/12 | - | - | - | - | - |
| **9 (55)** | LLLTeS | Turkey | Faculty members of educational institutions | - | - | - | 6/11 | - | - | - | - | - |
| **9 (56)** | LLLTeS | Turkey | Primary school teachers | - | - | - | 6/12 |  |  | - | - | - |
| **9 (57)** | LLLTeS | Turkey | Education- Prospective teachers | - | - | - | 12/12 | - | - | - | - | - |
| **9 (58)** | LLLTeS | Abu Dhabi | Education- Prospective teachers | 24/48 | - | - | - | - | - | - | - | - |
| **9 (59)** | LLLTeS | Turkey | Education- Prospective teachers | - | - | - | 9/12 | - | - | - | - | - |
| **9 (60)** | LLLTeS | Turkey | Faculty members of educational institutions | - | - | - | - | - | - | - | - | - |
| **9 (61)** | LLLTeS | Nigeria | Lectures & Undergraduate students | - | - | 10/12 | 12/12 | - | - | - | - | 11/12 |
| **9 (62)** | LLLTeS | Turkey | Education- Prospective teachers | - | - | - | 9/12 | - | - | - | - | - |
| **9 (63)** | LLLTeS | Turkey | Education- Prospective teachers | - | - | - | 6/12 | - | - | - | 14/28 | - |
| **9 (64)** | LLLTeS | Turkey | Education- Prospective teachers | - | - | - | - | - | - | - | - | - |
| **9 (65)** | LLLTeS | Turkey | Education- Prospective teachers | - | - | - | - | - | - | - | - | - |
| **9 (66)** | LLLTeS | Turkey | Education – English teachers | - | - | - | 9/12 | - | - | - | 16/28 | - |
| **9 (67)** | LLLTeS | Turkey | Education – Primary teacher | - | - | 9/12 | 12/12 | - | - | - | - | - |
| **9 (68)** | LLLTeS | Turkey | Education - teacher | - | - | - | 9/12 | - | - | - | 11/28 | - |
| **9 (69)** | LLLTeS | Turkey | Education -teacher | - | - | -- | 7/12 | - | - | - | 16/28 | - |
| **10* (70)** | Scale of Key Competence for LLL | Turkey | Education – Prospective teachers | - | 25/40 | 9/12 | 10/12 | - | - | - | - | - |
| **10 (71)** | SKCLLL | Turkey | University teachers | - | - | - | 10/12 | - | - | - | - | - |
| **10 (72)** | SKCLLL | Turkey | Education – Prospective teachers | - | - | - | 9/12 | - | - | - | - | - |
| **10 (73)** | SKCLLL | Turkey | Education – Prospective teachers | - | - | - | 7/12 | - | - | - | - | - |
| **10 (74)** | SKCLLL | Turkey | University teachers | - | - | - | 12/12 |  | - | - | 16/28 |  |
| **10 (75)** | SKCLLL | Turkey | Education - Mathematics Teachers | - | - | - | 9/12 | - | - | - | - | - |
| **11* (76)** | LLL competence scale | Cyprus | Education -Secondary school teachers | 39/48 | 32/40 | 8/12 | 12/12 | - | - | - | - | - |
| **11 (77)** | LLLCS | Turkey | Prospective teachers | - | - | 12/12 | - | - | - | - | 16/28 | - |
| **11 (78)** | LLLCS | Turkey | Primary school teachers |  |  | 9/12 | 12/12 | - | - | - | - | - |
| **11 (79)** | LLLCS | Turkey | Education -Secondary school teachers | - | - | - | - | - | - | - | 14/28 | - |
| **11 (80)** | LLLCS | Cyprus | Primary school teachers | - | - | - | 12/12 | - | - | - | - | - |
| **11 (81)** | LLLCS | Cyprus | Principals of primary schools | - | - | - | 10/12 | - | - | - | - | - |
| **11 (82)** | LLLCS | Turkey | Education – Physical education and sports (preservice) teachers | - | - | 7/12 | 12/12 | - | - | - | - | - |
| **12* (83)** | LLL Scale | Turkey | Education- Teachers | 10/48 | 20/40 | - | 7/12 | - | - | - | - | - |
| **13* (84)** | LLLS | China | Education- Teachers from universities | - | - | 8/12 | 9/12 | - | - | - | - | - |
| **14* (85)** | LLL Trends Scale | Turkey | Education- Prospective teachers | 27/48 | 30/40 | 12/12 | 12/12 | - | 4/8 | - | - | - |
| **14 (86)** | LLLTS | Turkey | Education- Prospective teachers | - | - | - | 12/12 | - | - | - | 12/28 | - |
| **14**  **(87)** | LLLTS | Turkey | Education- Prospective teachers | - | - | - | 9/12 | - | - | - | - | - |
| **14 (88)** | LLLTS | Turkey | Education – Primary school teacher | - | - | 6/12 | 9/12 | - | 3/8 | - | 16/28 | - |
| **14 (89)** | LLLTS | Turkey | Education – Teacher | - | - | - | 9/12 | - | - | - | 14/28 | - |
| **14 (90)** | LLLTS | Turkey | Education | **-** | - | 12/12 | 9/12 | - | - | 8/8 | 14/28 | - |
| **14 (91)** | LLLTS | Turkey | Education – school administrators; teachers | - | - | - | 7/12 | - | - | - | 16/28 | - |
| **15* (92)** | LLL Attitude Scale | Turkey | Education – Secondary school teachers | 33/48 | 28/40 | 11/12 | 10/12 | - | - | - | 9/28 | - |
| **16* (93)** | Coffelts’no name | US | Healthcare – occupational therapists | - | 20/40 | - | - | - | - | - | - | - |
| **17* (94)** | LLL Mindset | Canada | Professionals in accounting and finance |  |  | 10/12 | 10/12 | - | - | - | 11/28 | - |
| **18***  **(95)** | LLMS | Ethiopia | Education – Academic staff | 35/48 | 32/40 | 11/12 | 12/12 | - | - | - | - | - |

* Study in which an instrument to measure LLL is developed.

** Only relevance is assessed.

**Additional file 5: Information on the content and use of the instrument**

| **Instrument** | **Definition of LLL** | **Instrument’s content is based on** | **Type of items** | **Practical use** |
| --- | --- | --- | --- | --- |
| OCLI(12) | The perspective adopted in the present study, therefore, focused on the personality characteristics of individuals whose learning behavior is characterized by initiative and persistence in learning over time through a variety of learning modes, such as the modes of inquiry, instruction and performance proposed by Houle (1980). The term “self-directed continuing learning” was adopted to differentiate this broader view from “self-directed learning”, a term which is generally used in reference to a self-instructional process. | Input from experts  Learning theory (self-directed learning) | LLL attitude  Learning activities  Learning Skills | Screening tool to help educational programs select professionals for their programs |
| CLLP(15) | A highly trained person must constantly renew … knowledge. The goal is not merely to keep knowledge already acquired during the period of formal education. Much more than this – for past knowledge may be outdated – the aim is constantly to recharge the batteries that motivate and trigger self-renewal by keeping abreast of new knowledge that is constantly being added to by research and publication (Dubin, 1972). | Input from experts  Analysis of literature | LLL attitude  Learning activities  Learning skills  Personal characteristics | Screening tool to help educational programs select professionals for their programs  Tool that provides teachers and educators with insight into the LLL skills of their learners which helps them to adapt their teaching or use interventions in order to foster the development of these skills |
| SKILLS(19) | Learning strategies are the techniques or skills that an individual elects to use in order to accomplish a specific learning task. Learning strategies differ from learning style in that they are techniques rather than stable traits and they are selected for a specific task. Such strategies vary by individual and by learning objective. Often they are so customary to learners that they are given little thought; at other times much deliberation occurs before a learning strategy is selected for a specific learning task. | Learning theory (learning strategies) | Adult learning strategies | Individual learning tool  Tool that provides teachers and educators with insight into the LLL skills of their learners which helps them to adapt their teaching or use interventions in order to foster the development of these skills |
| CLLPS(21) | All-encompassing term that includes a variety of learning experiences which occur throughout an individual’s lifespan as the learner deliberately seeks to acquire knowledge, skills, attitudes and/or competencies relative to both personal and professional growth. | Previously developed instrument (CLLP)  Analysis of the literature | LLL attitude  Learning activities  Learning skills  Personal characteristics | Tool for curriculum evaluation and development |
| JSPLL(7) | We defined lifelong learning as a concept involving a set of self-initiated activities (behavioral aspect) and information-seeking skills (capabilities) that are activated in individuals with a sustained motivation (predisposition) to learn and the ability to recognize their own learning needs (cognitive aspect). | Analysis of the literature  Input from experts | LLL attitude  Learning activities | Tool for curriculum evaluation and development |
| ELLI(45) | The capacity and the desire to learn and to go on learning throughout life is now recognized as a central aspiration in the concept of `lifelong learning'.  This project began with a broader conceptualization of lifelong learning, drawing on research such as that by Smith and Spurling (1999) who aimed to define the reach and potential of real lifelong learning. They developed a holistic notion of lifelong learning that comprises two parts. Firstly, they suggest, lifelong learning relates to learning that takes place throughout the lifespan. Secondly, they suggest, lifelong learning includes the main types and classes of learning, both informal and formal education, as well as self-directed learning. Lifelong learning is relatively continuous, with a broad momentum that is maintained throughout life. It is intentional on the part of the individual or the  organization and is expressed in some form of personal or organizational strategy, formally or informally, which may be re-appraised over time. | Previously developed instrument  Analysis of the literature  Input from experts | LLL attitude  Learning activities | Tool that provides teachers and educators with insight into the LLL skills of their learners which helps them to adapt their teaching or use interventions in order to foster the development of these skills |
| WielkLLS(48) | We define lifelong learning from an individual’s perspective, following the definition of Hojat et al. (2006) that emphasizes the individual’s motivation to learn and the development  of associated skills to meet their own learning needs throughout the life span. | Previously developed instrument | LLL attitude  Learning activities | Tool that provides teachers and educators with insight into the LLL skills of their learners which helps them to adapt their teaching or use interventions in order to foster the development of these skills |
| Attributes of a Continuous Learner(53) | I have used the terms lifelong learning and professional development interchangeably. Ideally, professional development will lead to lifelong learning, particularly in the field of education. Professional development seeks to enhance lifelong learning by instilling the love of learning in educators. Professional development is an ongoing process of continuous improvement, not an isolated event or series of events. | Analyses of the literature | LLL attitude  Learning activities  Learning skills  Personal characteristics | Tool for curriculum evaluation and development |
| LLLTES(54) | Lifelong learning, is the lifelong, life wide, voluntary and self-motivated pursuit of  knowledge for either personal of professional reasons. As such, it not only enhances social inclusion, active citizenship and personal development, but also competitiveness and employability. The term recognizes that learning is not confined to childhood or the classroom, but takes place throughout life and in a range of situations. | Analyses of the literature  Input from experts | LLL attitude | Not specified |
| SKCLLL(70) | The European Union defined lifelong learning as ‘all the learning activities taken over in all parts of life within the perspective related to personal, citizenship and social affair in order to improve knowledge, ability and competence.’ (CEC, 2001a: 9) | Analyses of the literature  Input from experts | LLL attitude  Learning skills | Tool for curriculum evaluation and development |
| LLLCS(76) | ‘Lifelong learning’ policy allows them (learners) to exist as individuals who have access to information, use information in the proper place, learn how to learn besides being creative and critical thinkers. The concept of lifelong learning policy, which is understood as non-stop learning in our lives, is essential in satisfying the rapidly changing societal needs (Wang 2008) and following information and technology (Bryce 2004). Lifelong learning is an essential strategy (European Commission 2002). European Commission (2002) that province development and sustainability of information, skill and competencies of individuals (Cowan et al. 2004; Figel 2006; Sim et al. 2003). Most general sense, the lifelong learning approach is defined as ‘learning which has been acquired from cradle to tomb’ (Woodrow 1999). | Analyses of the literature  Input from experts | Learning skills | Not specified |
| LLLS(83) | Lifelong learning is an approach including knowledge, abilities and attitudes, as well as all of the learning activities of persons personal, social or employment-related (Önal, 2010). Lifelong learning education is carried on through profession-based education. Consistent with this, active citizenship, personal development, competitiveness and employability are also covered by philosophic field of lifelong learning and is continued throughout life (Aspin& Chapman, 2001). | Analyses of the literature | LLL attitude | Not specified |
| TLLLS(84) | Chan Lin (2013) pointed out lifelong learning as planned or unplanned learning activities, according to personal interests and needs, in the phases of individual life. Lifelong learning aimed to enhance an individual’s potential and fulfil the life ideal. Botha & Makoelle (2012) regarded lifelong and comprehensive learning being covered in lifelong learning, referring to all meaningful learning activities in various living environments in individual life, containing formal learning, non-formal learning, and informal learning, and aiming to enhance personal knowledge, affection, skills, and abilities to further enhance personal abilities of career development, life adjustment, and innovative responses and promote social progress and national development. | Analyses of the literature | Items not available | Not specified |
| LLLTS(85) | Lifelong learning is described as a continuous process and a multipurpose of learning activities taken with the objectives of improving one’s knowledge, skills and competence (OECD, 2001). Lifelong learning is a natural tendency to continue learning, growth and development and this trend is a process which may occur with the elimination of negative, insecure thoughts and belief systems, and the discovery of learning trends (McCombs, 1991). | Analyses of the literature  Input from experts | Items only available in Turkish | Tool that provides teachers and educators with insight into the LLL skills of their learners which helps them to adapt their teaching or use interventions in order to foster the development of these skills |
| LLAS(92) | In the continuously changing world, lifelong learning provides individuals with an opportunity to gain knowledge and competencies they need to be successful (Sharples, 2000). (…)  Dunlap and Grabinger (2003), likewise, in their studies, defined lifelong learning as conscious learning to increase both individuals’ life qualities and career developments. | Analyses of the literature  Input from experts | LLL attitude | Tool for curriculum evaluation and development |
| CNN(93) | If occupational therapists do not understand the process of continuing competence, they cannot effectively select professional development activities to develop their competence and learning. (…)  To promote ongoing professional development competence in health and practice areas, practitioners need to demonstrate a foundation of content and technical skills and develop their procedural knowledge of active listening and self-awareness by learning through observation, receiving feedback about how they interact with peers, and recognizing the role of contextual factors in the workplace (mentoring, case experiences, feedback, and support networks) (King, 2009; Lysaght et al., 2001).” | Analyses of the literature | LLL attitude  Learning activities | Not specified |
| LLLM(94) | Lifelong learning is the process of embracing learning throughout life (Candy et al., 1994). (….) There have been two dominant perspectives for discussing lifelong learning. The first could be called the system-level perspective. The system-level perspective addresses the systems, such as education institutions, that support lifelong learning (e.g., Aspin et al., 2001). The second perspective, the individual-level perspective, is concerned with the individual – the lifelong learner. | Previously developed instrument  Analyses of the literature | LLL attitude  Learning skills | Tool that provides teachers and educators with insight into the LLL skills of their learners which helps them to adapt their teaching or use interventions in order to foster the development of these skills |
| LLMS(95) | Lifelong learning is seen as one of the principles of the 21st-century global education paradigm. Education must be organized around four fundamental types of learning that will serve as the pillars of knowledge throughout a person’s life. These are learning to know, which is acquiring the instruments of understanding; learning to do, which is being able to act creatively in one’s environment; learning to live together, which is participating and cooperating with other people in all human activities and learning to be, which is an essential progression from the previous three. These pillars are not related to one specific phase of life. They complement one another throughout the life of individuals. | Analyses of the literature  Previously developed instruments  Input from experts | LLL attitude  Learning skills  Learning activities  Personal characteristics | Tool for educators and professionals who are working in HEIs to assess their lifelong learning dispositions. LLMS questionnaire could also be useful in evaluating the effectiveness of lifelong based learning/educational interventions. |
